# Leveraging sex-genetic interactions to understand brain disorders: recent advances and current gaps

**DOI:** 10.1093/braincomms/fcae192

**Published:** 2024-06-03

**Authors:** Nikita Neale, Frida Lona-Durazo, Mina Ryten, Sarah A Gagliano Taliun

**Affiliations:** Faculty of Medicine, Université de Montréal, Québec, H3C 3J7 Canada; Faculty of Medicine, Université de Montréal, Québec, H3C 3J7 Canada; Research Centre, Montreal Heart Institute, Québec, H1T 1C8 Canada; Department of Genetics and Genomic Medicine, Great Ormond Street Institute of Child Health, WC1N 1EH London, UK; Aligning Science Across Parkinson’s (ASAP) Collaborative Research Network, Chevy Chase, 20815 MD, USA; NIHR Great Ormond Street Hospital Biomedical Research Centre, Great Ormond Street Institute of Child Health, Bloomsbury, WC1N 1EH London, UK; Research Centre, Montreal Heart Institute, Québec, H1T 1C8 Canada; Department of Medicine & Department of Neurosciences, Faculty of Medicine, Université de Montréal, Québec, H3C 3J7 Canada

**Keywords:** brain, gene expression, genetic association, sex-bias

## Abstract

It is established that there are sex differences in terms of prevalence, age of onset, clinical manifestations, and response to treatment for a variety of brain disorders, including neurodevelopmental, psychiatric, and neurodegenerative disorders. Cohorts of increasing sample sizes with diverse data types collected, including genetic, transcriptomic and/or phenotypic data, are providing the building blocks to permit analytical designs to test for sex-biased genetic variant-trait associations, and for sex-biased transcriptional regulation. Such molecular assessments can contribute to our understanding of the manifested phenotypic differences between the sexes for brain disorders, offering the future possibility of delivering personalized therapy for females and males. With the intention of raising the profile of this field as a research priority, this review aims to shed light on the importance of investigating sex-genetic interactions for brain disorders, focusing on two areas: (i) variant-trait associations and (ii) transcriptomics (i.e. gene expression, transcript usage and regulation). We specifically discuss recent advances in the field, current gaps and provide considerations for future studies.

## Teaser

In this review, the authors provide an overview of recent advances and current gaps related to studying sex differences for brain disorders at the level of the genetic code and transcription. They offer considerations to move research forward to account for sex differences.

## Graphical Abstract

**Graphical abstract:**
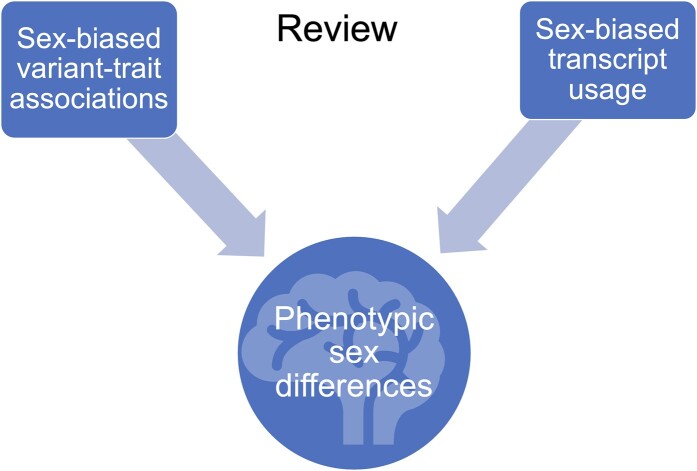

## Introduction

The concept that together sex (biological constructs) and gender (social constructs) (Box 1) are important modifiers of neuropsychiatric, neurodevelopmental and neurodegenerative diseases is becoming increasingly recognized, with genetic, biological, metabolic and environmental factors all contributing.^1-3^ Differences between the sexes occur at multiple levels (molecular, hormonal, anatomical, cultural, etc.), and a better understanding of the factors behind these differences will ultimately influence how care is delivered to affected individuals, impacting personalized and improved therapeutics and reducing the disparity in equity of care between the sexes. Indeed, multiple health care systems, including the UK National Health System, have adopted principles of equity of care.^4^ Nevertheless, despite some advances in sex-specific considerations, studies in this area are lagging in many domains, including but not limited to disorders of the brain with half of the population (females) being underrepresented in research studies.^5-7^ Disentangling these sex differences is challenging, but as molecular (genomics and transcriptomics) datasets increase in size, we assert the need and opportunity to better understand these differences starting at the level of DNA and RNA.

Box 1 **Clarification on the use of the term sex**

Here we use the term sex to describe the genetic sex karyotype of an individual, specifically the presence of two X chromosomes (‘female’) or one X and one Y chromosome (‘male’). We acknowledge that genetic sex is, however, a non-binary spectrum (for instance, individuals can have aneuploidy of the X or Y chromosomes or monosomy of the X), and that non-genetic factors such as gender-identity play important roles in complex human traits. These factors are beyond the scope of this review. Future work including individuals with non-XX/XY karyotypes and measures of gender-identity into genetic association analyses is warranted.

We note that while distinguishing the unique impacts of sex and gender on brain diseases is difficult given their interrelated nature, it is becoming clearer that across a range of conditions affecting the brain, there are significant differences in disease prevalence, manifestation and response to treatment between women and men influenced by societal factors often attributed to gender. Unipolar depression, for instance, is roughly twice as prevalent in women than men^8^ and tends to present with a different set of symptoms. Men tend to present with irritability, aggression, violence, substance abuse, risky behaviour, and somatic complaints, which are not so easily recognized as symptoms of depression by healthcare practitioners.^9^ In contrast, women seek treatment for depression, particularly during childbearing years, and are more likely to report hyperphagia, weight gain, hypersomnia, anxiety and greater severity.^10^ Furthermore, there is some evidence that women and men differ in response to treatment for depression with selective serotonin reuptake inhibitors being potentially more effective in women, whereas men tend to respond better to tricyclic antidepressants.^11^

While unipolar depression has been particularly well studied across the sexes, incidence and clinical manifestations, among other factors, can differ between females and males for a variety of central nervous system-related disorders.^12^ For example, autism spectrum disorder is more common in males than females with a ratio of 4:1 to 3:1 in favour of males,^13^ but these ratios may in part be due to affected females being missed by current diagnostic tests. Among neurodegenerative diseases, Parkinson’s disease, for example, affects males more than females, and there are differences between the sexes in presentation: depression and dyskinesias are more commonly observed in women compared to men, whereas rigidity and daytime sleepiness present more commonly in men than women.^14,15^ For Alzheimer’s disease, more females are affected, and again, the symptomology differs between the sexes, with higher depressive symptoms in diagnosed females compared to males and more agitation in diagnosed males than females.^16^

There are also established pharmacokinetic and pharmacodynamics differences between the sexes^17^ that can translate into differing biomarker ranges with implications for trial design.^17,18^ Focusing on the complement factors C4 and C3 proposed as schizophrenia biomarkers, it is recognized that at baseline they are present at higher concentrations in cerebrospinal fluid and plasma in males (aged 20–50 years) as compared to females.^19^ Given biomarker experience in other fields, that have incorporated sex-specific knowledge with routine clinical implications, such as having sex-specific normal ranges for estimated glomerular filtration rate to assess kidney health^20^ and accounting for sex in the Framingham risk score as a metric of cardiovascular health,^21^ we anticipate many more examples of this kind to emerge across neuropsychiatric and neurodegenerative diseases.

Taken together, and despite the limitations of existing studies of sex differences across neurological and neuropsychiatric disease, it is evident that they could be driven by a combination of genetic,^3,22^ hormonal and environmental differences that aggregate over time. Determining the relative importance of these factors is key because of the implications for our understanding of pathophysiology and the identification of effective therapies. Of these factors, facilitated by the availability of large-scale data and analytical means, genetics presents a promising avenue of research. Differing genetic architectures between the sexes could partially explain phenotypic differences, and thus an increased understanding of the molecular basis of sex-genetic interactions will be crucial to move towards the identification and development of treatment options that are tailored to the sexes. Although sex-specific research for brain disorders at the molecular level has commenced, it is far from being fully explored. There is a critical need for work in this area to move forward, and so we aim to raise the profile of this field as a research priority. Therefore, the goal of this review is to summarize the importance of investigating sex-genetic interaction for brain disorders by highlighting recent advances in the field and current gaps. Where possible, we also provide concrete considerations or solutions to move forward.

**Figure 1:**
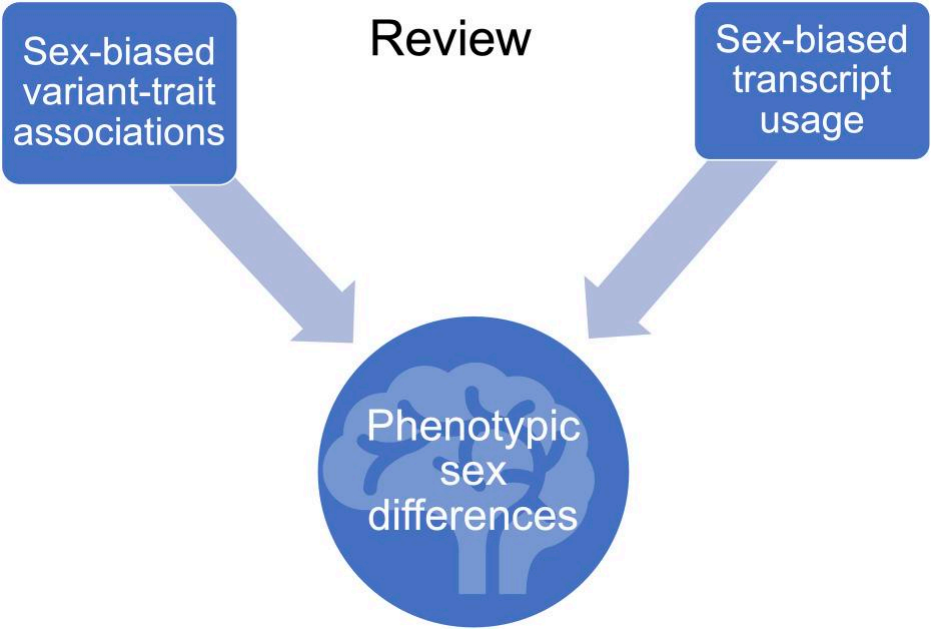
**Visual representation of the aim of this review**. Here we summarise sex-biased variant-trait associations and gene expression to better understand phenotypic sex differences in brain disorders.

Our focus here will be on sex-genetic interactions at the following two levels (i) variant-trait associations and (ii) transcriptomics encompassing sex-biases in gene expression, transcript usage and regulation (Fig. 1). Specifically, we focus on how an increased understanding of sex-genetic interactions at the molecular level can enable the identification of novel gene targets and/or biological pathways. This knowledge will ultimately inform a personalized approach to medical care where differences between females and males are not only acknowledged but used to advance care. Future work will be needed to disentangle the impact of sex and gender on disease risk, including the identification of phenotypic outcomes arising from primarily gender-related societal and cultural exposures as opposed to genetic sex.

### Identifying sex-genetic interactions through genome-wide association studies in datasets of increasing size

Genome-wide association studies (GWAS) detect genomic risk loci that are associated with a change in the trait of interest by testing for differences in effect allele frequencies. GWAS have been key to identifying variant-trait associations for common complex human traits, and large sample size is a primary factor for the detection of associations with small effect sizes, typical of variants associated with complex traits.^22,23^ Importantly, some risk loci, termed sex-biased loci, may differ between the two sexes. A locus could be strongly associated with the trait of interest in one sex, but with no evidence for association in the other sex, or in the most extreme cases have an opposing direction of effect between the sexes. More often seen in current data (though this may change in the future with increased sample sizes), are variants with the same direction of effect but with either similar or differing magnitudes in males and females. Sex-biased risk loci can be detected most reliably through conducting statistical tests for association in female-only or male-only individuals from the study population and then carrying out tests of heterogeneity to assess significantly different association signals between the sexes. These sex-stratified analyses inevitably require larger sample sizes compared to non-sex-stratified analyses to detect variant-trait associations, and so have been largely de-prioritized. However, advances in genotyping technology and the collaborative nature of recent large-scale cohorts and biobanks have helped to increase the number of individuals available for GWAS and open the possibility of sex-stratified GWAS for a variety of complex traits, including disorders of the brain. For instance, genotype and various phenotype data are available through biobanks such as the UK Biobank, Biobank Japan, China Kadoorie Biobank, and the NIH All of Us Research Program,^24-27^ as well as other large-scale initiatives such as the NHLBI Trans-Omics for Precision Medicine program, TOPMed and the Psychiatric Genomics Consortium.^28,29^ Sex-stratified genome-wide summary statistics in the UK Biobank, for example, have been made available for a wide set of traits.^30^ Additional examples are outlined below.

There is a clear role for the sex chromosomes in driving sex-genetic interactions in brain disorders, with an overrepresentation of genes expressed on the X chromosome involved in brain function.^31^ Autosomal variation can also influence sex-genetic interactions, and current data available suggests that sex-specific genetic effects across polygenic diseases may be small and dispersed throughout the autosomes. Martin *et al*., for instance, examined sex differences in genetic effects of autosomal variants with a minor allele frequency greater than 1% across several neuropsychiatric and behavioural traits and identified statistically significant differences only among analyses with the largest sample sizes, such as ADHD, lifetime cannabis use and schizophrenia.^32^ In the case of lifetime cannabis use, the authors showed that by performing a sex-stratified GWAS, they observed stronger effect sizes in females than in males, albeit not genome-wide significant, even though the case female:male ratio was close to one.^32^ These results support the need of performing sex-stratified GWAS in future work, as sample sizes continue to increase to reach sufficient power to detect potentially small sex-specific effects.

Identifying which risk loci show sex-biases, whether they are on the sex chromosomes or on the autosomes, can help us begin to better understand the molecular mechanisms driving the various sex-biases observed across various neuropsychiatric, developmental and neurodegenerative disorders. Variants with two alleles present in both sexes (e.g. autosomal variants) present the simplest case for analysis, as they are equally analysed in males and females. Then there is the scenario where an allele is only present in one sex, but not in the other, as is the case for Y chromosome variants, for which there is no X homologue and are only present in males in a hemizygous (i.e. one allele) state. Finally, a more complicated scenario is where there is variability in expressed alleles, such as on the X chromosome, where there is inactivation and escape from inactivation. Evidence for sex biases at the level of genetics or the lack of such evidence both provide useful information. A lack of evidence, for example, could imply that either sex-environment interactions are the major contributors at play, or that larger sample sizes are required to increase power to detect sex-genetic interactions with smaller effect sizes.

#### Statistical power

**Figure 2:**
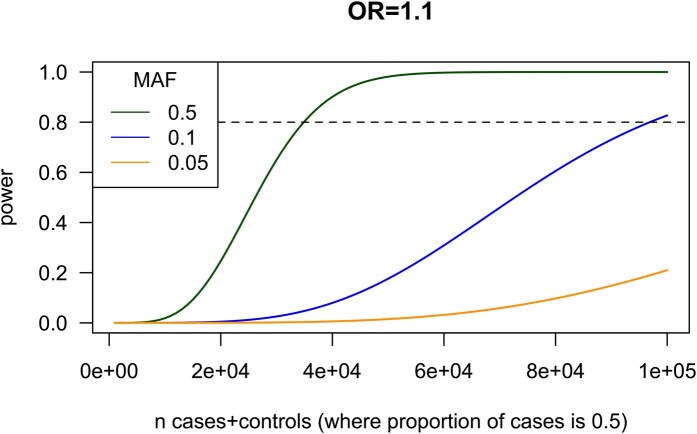
**Power curves at varying sample sizes.** Power curves corresponding to three genetic variants each with an odds ratio (OR) of 1.1 with different minor allele frequency (MAF) for a binary trait.

Statistical power to detect associations between a genetic variant and a binary trait depends on numerous factors, including the effect size, minor allele frequency of the genetic variant and the proportion of cases to controls. Sex incidence biases, which are often seen in disorders affecting the brain, can present additional challenges (Box 2). Current sample sizes can permit over 80% power to detect common variants even when splitting the sample into male and female sub-groups, depending on disease prevalence and variant effect sizes. However, much larger sample sizes are required to detect rarer variants with modest effects or common variants with small effects. For instance, the sample sizes required for 80% power to identify a significant association (*P* < 5 × 10^−8^) of a variant with an odds ratio (OR) of 1.4 are 6,400, 54,000, and 540 000 for a minor allele frequency (MAF) 0.1, 0.01, and 0.001, respectively (assuming 5% disease prevalence).^33^ At smaller effect sizes (e.g. OR = 1.1), even larger sample sizes are required for sufficient power. For example, in this scenario, a total sample size for a female-only (or male-only) analysis of close to 100 000 (of which 50 000 are cases) would be required to reach 80% power to detect an association with a variant with MAF = 10%. This total sample size of 100 000 yields only around 20% power to detect a variant with the same effect size (OR = 1.1) but a lower frequency (MAF = 5%) (Fig. 2).

Box 2 **Additional challenges to statistical power presented by sex incidence biases**

Case misclassification (labelling a participant who is a case as a control) will negatively impact power. When misclassification is more likely to occur in one sex than the other, for instance, in scenarios where female cases are more often mis- or under-diagnosed compared to males, or vice versa, the misclassification problem will be skewed. The result is differential power in the male- and female-specific analyses even if all other criteria (sample size, effect size, etc.) remain constant.

Similarly, the issue of mis- or under-diagnosis in one sex or the other can result in sex-biases in power even when case/control numbers are similar. In addition, the different symptomology could mean that even when the diagnosis is accurate, the disease stage between the sexes at the time of diagnosis could differ. This scenario could result in the recruitment of cases at a more advanced or severe disease stage in one sex compared to the other. Such an outcome is particularly important for potential biases in genetic association analyses aimed at assessing disease progression, such as in the context of neurodegeneration.

#### Study design considerations for sex-genetic interactions

The collection of study participants for GWAS, whether it is a cohort (case/control) design or a consortium-style recruitment, such as access to electronic health records for research use, in which cases and controls are defined post hoc, have biases that must be considered when running the association analyses and in the interpretation of the results. Selection bias, survivor bias, access to health care, geographical location and other factors can skew association results leading to false positive or false negative associations.^34,35^

Analyses aimed at assessing sex-genetic interactions pose additional considerations. For instance, for binary traits or diseases in which there is a sex bias in disease prevalence, an unequal sex distribution in the case arm could result, thus requiring the use of statistical approaches that allow for case-control imbalance. Additionally, diagnosis is also an important consideration, particularly for disorders of the brain. First, the ability to diagnose well in both sexes, as there could be a lack of understanding of symptoms in one sex due to lack of study, which could also lead to recruiting cases at a difference disease stage in one sex compared to the other. Second, possible unequal access to diagnosis due to societal factors and/or stigma preventing one group from seeking medical advice more than another. One such example is the high stigma that males with eating disorders encounter, in addition to the stereotypes associated with eating disorders, which can lead to under-diagnosis and consequentially under-treatment.^36,37^ To summarize, the availability of a large sample with unbiased diagnoses across the cohort/consortium is optimal. However, performing genetic associations while considering the factors presented above would similarly contribute to the advancement of the field.

#### Current state of the field for sex-stratified and sex-chromosomes association analyses

##### Autosomal genetic variant-trait associations

At autosomal variants, individuals carry two alleles regardless of sex (assuming no autosomal aneuploidy). In GWAS, sex is often included as a covariate (regressed out in the model), but this technique does not permit the identification of sex-biased results, as it is done precisely to mask the confounding effects between the sexes. In recent years, larger sample sizes, which increase statistical power, have been allowing for the possibility of conducting sex-stratified analyses, including for disorders of the brain, although this has been performed for a limited number of phenotypes. Examples include sex-stratified GWAS results in the UK Biobank for depression^38^ and neuroticism^39^ and for schizophrenia in work done by the Schizophrenia Working Group of the Psychiatric Genomics Consortium.^40^ There are also sex-stratified results for autism spectrum disorders,^41^ Parkinson’s disease^42^ and endophenotypes for Alzheimer’s disease.^43-45^ Performing sex-stratified GWAS can be done using a similar pipeline as in a sex-combined analysis, but analyses are performed separately in each sex, and therefore sex is not included as a covariate. Assuming diagnoses to be accurate in both sexes, the main current obstacle is obtaining large enough sample sizes in each group to achieve adequate power to identify effects that may (or may not) be shared between sexes through tests of heterogeneity.

##### Chromosome X genetic variant-trait associations

Among the examples of sex-stratified analyses available for brain disorders that we are aware of, interestingly, several only assessed autosomes (Table 1). The lack of chromosome X even in studies aimed to identify sex differences, emphasizes the complexity of chromosome X analysis even within one sex, including lower genotype imputation in the pseudo-autosomal regions (PAR1 and PAR2) and the lack of scalable and standardized ways to account for X-inactivation for female-specific analyses. There are several characteristics specific to the X chromosome that make it unique to autosomes and thus pose analytical challenges in association testing. These challenges extend to impeding the carrying out of gene expression analyses as well (see next section). Such challenges include X-inactivation in females (Box 3), the hemizygous state of genotypes in males and PAR1 and PAR2 on either chromosomal end (homologous to regions on the human Y chromosome). Limited large-scale studies investigating X-inactivation in complex traits have been conducted.^47^ Despite these challenges, the relative size and number of genes X contains relative to the autosomes, make a convincing argument to include the X in association testing (Box 4).

**Table 1:** Non-comprehensive list of examples of published studies of brain disorders for which sex-stratified genome-wide association study (GWAS) results are available

GWAS	*N* cases	*N* controls	Chromosomes tested	Reference
Alzheimer’s disease endophenotypes (cerebral spinal fluid levels of amyloid-β 42 (Aβ42) and tau)	1527 males and 1509 females		1-22	Deming *et al*. 2018^44^
Alzheimer’s disease endophenotypes (autopsy measures of amyloid-β 42 (Aβ42) and tau)	Up to 2701 males and 3275 females		1–22	Dumitrescu *et al*. 2019^43^
Autism Spectrum Disorders	1546 males and 338 females	492 female controls and 1012 males	1–22, X	Mitra *et al*. 2016^41^
Depression	34 923 males and 62 066 females	92 944 males and 84 208 females	1–22	Silveira *et al*. 2023^38^
Parkinson’s disease	13 020 males (+ 7936 paternal proxy cases) and 7947 females (+5473 maternal proxy cases)	89 660 males and 90 662 females	1–22	Blauwendraat *et al*. 2021^42^
Neuroticism	129 229 males and 145 669 females		1–22, X	Wendt *et al*. 2023^39^
Schizophrenia	18 346 males and 9837 females	17 122 males and 16 763 females	1–22, X	Blokland *et al*. 2022^40^

Box 3 **X inactivation in females**

As a compensatory mechanism to account for the double dosage of genes in females carrying two X chromosomes compared to males with only one such chromosome, certain genes are inactivated or not expressed in one copy in females. Which gene is inactivated can be random, but there is also evidence that the choice can happen in a non-random manner, with evidence that imbalanced expression of the paternal and maternal X chromosomes is common in the general population.^46^ Genes can also escape inactivation, which can manifest differently depending on cell type.

Box 4 **Characteristics of the human X chromosome relative to the 22 autosomes and chromosome Y**

Compared to the autosomes, there is evidence that chromosome X is under recent positive selection.^48,49^ There is a higher density of brain-expressed genes on the X.^50,51^ The human chromosome X is the sixth longest chromosome in terms of base pair length. It is most similar in length to chromosome 7. Of the chromosomes, the X is ranked fourteenth in terms of the highest number of protein-coding genes (as defined in Gencode, version 35), containing a similar number of genes as chromosome 8. In contrast, the Y chromosome contains <30 genes. An OMIM search (conducted 29 August 2023) for chromosome X regions, presented a total of 362 phenotype-only entries on this chromosome with a variety of inheritance patterns (including X-linked, X-linked recessive, X-linked dominant, pseudo-autosomal recessive and pseudo-autosomal dominant), many of which are linked to brain disorders.^52^ Indeed, a relatively higher number of Mendelian diseases are associated with X chromosome compared to the autosomes.^53^

**Figure 3:**
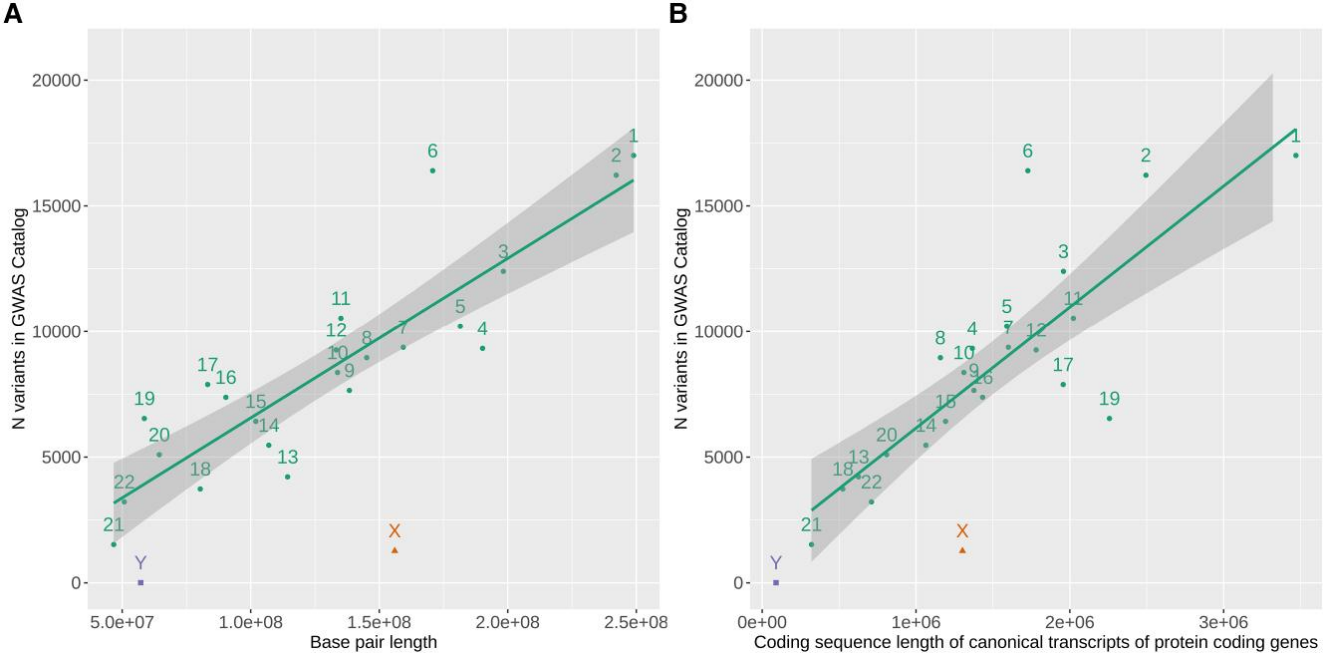
**Number of reported variants (unique base pair positions) present in the EBI-NHLBI GWAS catalog (last updated 2023-06-22) split by chromosome base pair length (A) or coding sequence length of canonical transcripts of protein-coding genes (B).** The coding sequence length is based on data from Gencode version 44. The line is the regression line for autosomal data, and the shaded area around it represents the 95% confidence interval. The autosomes are represented by circles. The X chromosome is represented by a triangle and the Y is represented by a square.

Compared to the autosomes of similar size, the number of variant-trait associations identified on the sex chromosomes is limited, relative to their length, which is likely due to the lack of inclusion of the sex chromosomes in association studies (Fig. 3). Although chromosome X was sequenced around 20 years ago,^53^ this chromosome is still not routinely assessed in genetic association studies. A call for inclusion of chromosome X into genome-wide association analyses was presented in 2013, in which a survey of published genome-wide association studies from 2010 and 2011 showed that only 33% had tested variants on the X chromosome.^54^ More recently, a similar analysis was conducted. Of the 136 publications that submitted at least one summary statistics file to the NHGRI-EBI GWAS Catalog in 2021, only 25% reported chromosome X association results suggesting that, if anything, fewer studies had included the X chromosome in their analyses.^55^ This proportion is despite improvements in the quality of the sex chromosome builds. Only recently, a telomere-to-telomere assembly for the X chromosome was released, which helped fill in gaps in the reference genome for this chromosome.^56^ This sequence, in addition to the telomere-to-telomere assembly 62 million base pairs in length for the Y chromosome^57^ will be particular useful to accurately characterize the challenging pseudo-autosomal regions (PAR1 and PAR2).

Indeed, recent studies that have included the X-chromosome are yielding robust, significant results. A recent X-chromosome-wide association study for Parkinson’s disease risk in individuals of Latin American genetic ancestry, identified eight associated regions, including a previous chromosome X finding from studies in European genetic ancestry individuals.^58^ Additionally, a GWAS of multiple sclerosis, a neurodegenerative and autoimmune disease with a higher incidence in females, identified a genome-wide significant signal on the X chromosome that lies on a T cell enhancer peak.^59^ In contrast, a trans-ancestry GWAS of schizophrenia did not find significant signals on the X chromosome,^60^ although limitations on statistical modelling for variants on this chromosome (see below) could be contributing.

Although some statistical tools provide recommendations for chromosome X association analyses, there are no standards, and the way to code alleles for association testing is variable (Supplementary Table 1). To our knowledge, currently, there is no widely accepted statistical method that accounts for X-inactivation in association testing, but methods have been proposed with different models (random X inactivation, escape of X inactivation or skewed X inactivation).^61-64^ Similarly, there are no widespread guidelines on how to handle variants falling into PAR1 and PAR2 versus variants that fall outside these regions, and some tools, including those specific to X chromosome association analyses, remove variants in the PAR1 and PAR2 regions as a quality control step.

The commonly used tool PLINK, offers two ways in which to perform chromosome X association tests: a female-only analysis or using all samples in the regression, but including sex as a covariate.^65^ In the analysis including both sexes, males should be coded as diploid (i.e. male genotypes are coded as either 0/2), and female genotypes are coded as 0/1/2. The latter corresponds to a model of random X inactivation, whereby two copies of the X chromosome in females do not have twice the effect of a single copy of the X in males. However, random X inactivation may not be realistic given the evidence that skewed X inactivation is associated with age, which can impact disease risk.^54,66-69^ Besides, skewed X inactivation could also occur due to inheritance of particular alleles, at least for rare variants.^46^ As for coding of the pseudo-autosomal regions of chromosome X, PLINK chooses to represent them as a separate ‘XY’ chromosome. This convention is to avoid special handling of male chromosome X heterozygous calls, and the software has special flags to format the input data. SAIGE and Bolt-LMM^70,71^ do not handle chromosome X differently from autosomes, and Regenie^72^ requires males to be coded as diploid. SAIGE, Bolt-LMM and Regenie do not code variants falling within the pseudo-autosomal region differently from the variants in the rest of chromosome X. These commonly used tools do not currently allow for modelling escape from X inactivation or skewed X inactivation.

##### Chromosome y genetic variant-trait associations

Chromosome Y is substantially smaller than the X, and in evolutionary time has become shorter, with gene decay. Nevertheless, the Y contains important genetic information for male development and function, particularly the region encoding the testis-determining factor, the *SRY* gene. For instance, it has been shown in animal and cell culture models of Parkinson's disease, a disease with higher incidence in males, that suppression of *SRY* expression diminished neurodegeneration and motor deficits.^73^ On this chromosome, even variant calling itself is challenging for numerous reasons, such as due to the high content of repetitive sequences and segmental duplications,^74^ which has certainly hindered the study of chromosome Y variants in association studies. In the human Genome Reference Consortium (GRCh) version 38 sequence, more than half of the Y’s sequence is absent, and the most recent complete sequence of this chromosome carried out by the Telomere-to-Telomere Consortium corrects multiple errors in the GRCh38 chromosome Y sequence. The Y chromosome has largely been omitted from large-scale GWAS.^57^ Some existing association tools can support Y association testing, but standards on how to carry out association testing on chromosome Y vary among software (Supplementary Table 1). Additionally, it is possible to perform associations at a single-variant level or at the level of haplogroups, given the high LD in the male-specific region of the chromosome.^75^ Multiple obstacles have hindered the inclusion of this chromosome, including the lack of coverage and accuracy across the chromosome, statistical tools designed to specifically handle chromosome Y, and statistical power to detect small effect sizes when restricting available samples to males only. One study of Parkinson’s disease explored the association with Y chromosome haplogroups and single Y chromosome variants, albeit they did not find significant associations, which the authors note could be in part due to a large portion of the chromosome (30 Mb) being difficult to assess given its heterochromatic nature and the presence of repeats.^76^ Similarly, no significant associations with multiple sclerosis have yet been identified with more recent Y chromosome data.^59^

#### Considerations surrounding sex-stratified genetic associations

As with any association test, it is essential to acknowledge that GWAS findings point to correlation and not necessarily causation. Indeed, a causal inference approach making use of genetic variant-trait association statistics and given certain assumptions (Mendelian randomization) has become immensely popular in recent years to dissect potential causal relationships between an exposure (e.g. a modifiable risk factor, gene expression levels, etc.) and a disease of interest.^77^ This approach can use sex-stratified GWAS association results for the exposure and outcome to interrogate potential sex-biases in terms of causal effects. Furthermore, association results cannot discredit other exposure by sex interactions as drivers of the observed signal. Careful downstream functional follow-up is needed to test causality. Furthermore, associations discovered in one genetic ancestry group may not be transferable to other groups due to differences in allele frequencies and correlation among nearby genetic variation due to selective factors in recent human demographic history or to differing effect sizes. Additionally, loci displaying sex-bias may only be observed in certain environmental conditions, pointing to gene-by-environment (by sex) interactions. Gene-by-environment interactions are challenging to disentangle from sex-genetic interactions and require much greater samples sizes. For instance, assuming that the effect of the interaction term is half of that of the main terms, approximately four-times the sample size is needed to reliably detect the interaction effect. Finally, additional influencing factors are likely to play a role, including epigenetic modifications, which are changes to chromatin structure that do not modify the actual nucleotide sequence, or environment-induced epigenetic modifications. As sample sizes for brain disorders continue to grow, future work on these additional contributors and gene-by-sex interactions is warranted.

#### To the future

We strongly encourage analyses of the sex chromosomes to foster sex-genetic discoveries for complex human traits of the brain. Efforts are being made to conduct sex-stratified GWAS, such as in the UK Biobank,^30^ but this direction of work is still limited for consortium-scale efforts for brain-related traits. GWAS on autosomes is routinely conducted and analytical standards and well-used software exist.^78,79^ However, the sex chromosomes should not necessarily be analyzed the same way as autosomes due to biological differences that make these chromosomes unique from autosomes, including the hemizygous state of males for chromosome X sites and X inactivation in females. The lack of recombination on the Y chromosome (apart from recombination between the two small paralogous regions at either end of the X chromosome), which results in large stretches of linkage disequilibrium pose additional analytical challenges for association analyses on this chromosome that warrant a different approach than used for the autosomes. Thus, the development of standardized sex chromosome analysis recommendations and guidelines (for both X and Y), followed by implementation of either easy-to-use and intuitive software or extensions to popularly used tools to apply these strategies must be pursued to achieve this need and make association analyses on the sex chromosomes accessible and subsequently the standard. An excellent step forward in this direction is a recent publication providing concrete guidelines on how to perform sex-aware genetic analysis, including quality control steps.^80^

### Sex-genetic interactions in the context of transcriptomics

Large-scale datasets containing post-mortem brain samples derived from males and females have empowered studies investigating sex-biases at the transcriptomic-level, including differential gene expression, co-expression, splicing, and transcriptional regulation (Table 2). The Genotype Tissue Expression (GTEx) Consortium, PsychENCODE^92,93^ and the CommonMind Consortium,^94,95^ for instance, have each collected post-mortem brain samples for which genotypes, RNA-sequencing data and sex information are available. These resources are and will continue to be pivotal for increasing our understanding of the role of sex in brain disorders. However, there are limitations: post-mortem brain tissue collections remain comparatively small, and there is a skew towards the collection of male samples, so significantly limiting the interpretability and generalisability of results. Here, we will discuss current knowledge on the potential roles of sex-biased gene expression/co-expression, transcript usage, and expression quantitative trait loci (eQTL). We will conclude with a summary and suggestions for directions for future research.

**Table 2:** Overview of examples of transcriptomics datasets in brain tissue that have been used to study sex-genetic interactions in the context of gene expression

Dataset	Example paper using dataset	Tissue types	Age range of samples	Sample size			Genetic ancestry of samples
				*N* Female	*N* Male	Total	
BrainSpan; Miller *et al*., 2014^81^	Shi, L. *et al*., 2016^82^	CNS post-mortem	8–24 wkspc	4–8	4–9	8–17	Varied (European, Asian, African, Hispanic)
			4 months–4 years	2–3	3–6	5–9	
			8–19 years	2–3	2–4	4–7	
			21–40 years	2–3	2–3	4–6	
Brain Span; Kang *et al*., 2011^83^	Kang *et al*., 2011^83^	CNS post-mortem	5 wkspc-82 years	26	31	57	Varied (European, Asian, African, Hispanic)
UKBEC; Trabzuni *et al*., 2013^84^	Trabzuni *et al*., 2013^84^	CNS post-mortem	M: 57 (16–91) years F: 64 (20–102) years	36	101	137	European
GTEx v6p; Ardlie *et al*., 2015^85^	Lopes-Ramos CM *et al*., 2020^86^	29 post-mortem tissues	18+	188	360	548	Varied (European, Asian, African, Hispanic)
GTEx v8; Aguet *et al*., 2020^87^	Oliva, M. *et al*., 2021^88^; Aguet *et al*., 2020^87^	44–49 post-mortem tissues	18+	281	557	838	Varied (European, Asian, African, Hispanic)
ROSMAP^89^	Xia *et al*., 2021^90^	CNS post-mortem	65+	471*	227*	698*	Varied (European, African, Hispanic)
DBCBB^91^	Labonté *et al*., 2017^2^	CNS post-mortem	18+	22	26	48	European (French Canadian)

Abbreviations: GTEx = Genotype Tissue Expression Project; UKBEC = UK Brain Expression Consortium; ROSMAP = Religious Orders Study/Memory and Aging Project; DBCBB = Douglas Bell Canada Brain Bank; wkspc = weeks post-conception; * = sample sizes of a subset of nonpsychiatric controls used in Xia *et al*.^90^

#### Autosomal genes as a source of sex-biased gene expression in human brain

Autosomal genes exhibiting differential expression levels between the sexes may explain some of the observed sex differences observed in brain conditions. Indeed, a recent analysis conducted by the GTEx Consortium identified 1559 sex-biased genes located on autosomes, and the presence of sex-biased autosomal genes is also supported by other studies.^83,84,88^ Interestingly, using a positional gene set enrichment analysis method and based on male- and female-biased genes from across all human tissues studied, there is evidence to suggest that genes with sex-biased expression are enriched not only within 5 X-linked regions but also within 134 autosomal regions. What is more, in work conducted using the UK Brain Expression Consortium dataset analysing 12 brain regions from adult human brains, 34% of all genes with sex-biased gene-level expression were located on the autosomes.^84^ Previous work in other cohorts have also identified sexually dimorphic autosomal gene expression in human brain,^83,96^ highlighting the importance of assessing autosomes to further our understanding of sex differences in brain disorders.

Two studies using RNA-sequencing data spanning the stages of human brain development and based on the BrainSpan cohort, suggest that the expression of sex-biased genes depends on developmental stage and agrees on the key role of sex chromosomes and autosomal genes.^82,83^ More specifically, these studies suggest that prenatal stages of brain development are characterized by higher sex-biases in gene expression, in terms of the number and magnitude of sex effects, while adulthood was the period with the smallest number of differentially expressed genes.^83^ Furthermore, one study found regional sex-biased differences, with more male-biased gene expression across most brain regions, except for the amygdaloid complex, the mediodorsal nucleus of thalamus, striatum and primary visual cortex.^82^ Interestingly, this study noted significant shifts in sex-biased gene expression both in early childhood and at puberty. The former was characterized by more female sex-biased gene expression across most brain regions, whereas the latter was characterized by more male sex-biased gene expression.^82^

In this context, the identification of female-biased gene expression is particularly restricted by the age of the individuals for which samples have been collected, and by the absence of relevant clinical information on their hormonal status. Adult female brain tissue is most commonly derived from post-menopausal females, limiting gene expression and transcriptional knowledge in pre-menopausal females. Assaying gene expression in post-menopausal females limits current knowledge of female-biased expression at life stages where a stronger influence of hormonal states may lead to increased sex-biased effects. Indeed, many genes across the genome have hormone-sensitive promoters, and thus levels of certain hormones, such as estrogen, influence expression levels.^97^Additional data on the hormone status of female samples from brain banks (including menarche/menopause status as a metric of estrogen exposure, use of oral contraceptives and hormone replacement therapy, etc.), or validated tools to infer hormonal status are required to better interpret sex-biased differences throughout the lifespan. Such data are not routinely collected in the context of these studies but are likely to contribute to the interpretation of results. Information on hormones will be needed to better distinguish hormonal–genetic interactions from sex-genetic ones, as well as understanding the role of hormones in genetic regulatory mechanisms.

Since most of the spatio-temporal regulation of protein-coding genes in the brain occurs during prenatal development and puberty, these findings could explain the early sexual divergence of human brain^83^ and differences in susceptibility to disease across the sexes. In fact, analyses of the BrainSpan samples, assessing genes on either the autosomes or chromosome X at these developmental stages, suggested that genes causally associated with autism, obsessive-compulsive disorder, major depressive disorder, bipolar disorder, schizophrenia, Alzheimer’s and Parkinson’s were more highly expressed in males.^82^ In contrast, genes highly expressed in females were enriched for those causally associated with obsessive-compulsive disorder, schizophrenia, epilepsy and Alzheimer’s, with notable differences across developmental stages.^82^ Additionally, within the UK Brain Expression Consortium (UKBEC) and BrainSpan cohorts, several genes that exhibited sex-biased regulation, either through alternative transcript usage or expression-levels, were found to be associated with neurological conditions, including those with known sex differences in incidence or progression, Table 3.​^81-83^ ​Therefore, differences in gene expression between the sexes may account for some of the observed clinical differences in brain phenotypes and diseases.^83^

**Table 3:** Examples of genes with evidence of sex-biased expression associated with brain disorders

Gene	Region of biased expression	Developmental stage of samples	Male or female-biased expression	Description of associated disorder(s)	Study
*CD24*	Cerebellum	Age range of 16–102 years	Male-biased	Multiple sclerosis	Trabzuni *et al*., 2013^84^
*RSPO1*	Hypothalamus	Age range of 16–102 years	Male-biased	Sex-reversal phenotype in females	Trabzuni *et al*., 2013^84^
*S100A10*	Brain	Age range of 5 weeks post-conception-82 years	Male-biased	Depression	Kang *et al*., 2011^83^
*IL33*	Brain	All four major developmental stages studied (prenatal, early childhood, puberty and adulthood)	Male-biased	Improvement in Alzheimer's-like symptoms	Shi *et al*., 2016^82^
*APLNR*	Brain	All four major developmental stages studied (prenatal, early childhood, puberty and adulthood)	Male-biased	Downregulation led to sub-chronic variable stress (mice models)	Shi *et al*., 2016^82^
*GPR37*	Brain	All four major developmental stages studied (prenatal, early childhood, puberty and adulthood)	Female-biased	Anxiety and depression-like symptoms in female mice (mice models)	Shi *et al*., 2016^82^

#### Genes located on the X and Y chromosome and sex-biased gene expression

Genes located on the X and Y chromosomes are an evident potential source of sex biases in gene expression for brain disorders with many X chromosome genes in particular known to contribute to brain diseases. Of the genes in the OpenTargets Platform^98^ causally associated with a rare brain disease (searched October 27, 2023, and with a ClinVar evidence score ≥0.7 associated with EFO_0005774), 83 (13%) are located on the X chromosome compared to 550 genes on the autosomes. Not only are X chromosome genes over-represented, but they can give rise to phenotypic variability in disease severity across the sexes. Since females inherit two copies of the X chromosome and males only one, it is most often the latter that express a more prominent disease phenotype, with females spared from the most severe manifestations. One example of such an X-linked disorder is Fragile X syndrome, which is most commonly caused by (CGG)*n* repeat expansions of >200 repeats in the *FMR1* gene located on the X chromosome.^99^ However, X-linked disorders are not always more severe in males as in the case of developmental and epileptic encephalopathy type 9 (also known as *PCDH19* epilepsy). *PCDH19* epilepsy is caused by loss-of-function pathogenic variants in the gene *PCDH19,* which encodes for a protocadherin involved in cell-to-cell adhesion.^100^ Several hypotheses have been posed to explain why *PCDH19* epilepsy is linked to females and not males. One hypothesis being that hemizygous males have a population of *PCDH19*-negative cells, whereas carrier females are likely mosaics, and the existence of both *PCDH19*-negative and *PCDH19*-wild-type cells may interrupt cell-to-cell communications resulting in the observed phenotypes in females.^100^ Other possibilities are a functional rescue or compensatory factor in males, a dominant negative effect in females or sexual dimorphism in expression or function of *PCDH19*.^100^ All possibilities hint at the complexity of X chromosome gene expression and regulation in human brain across the sexes.

To date, analyses of differential gene expression between the sexes in the human brain have highlighted genes of the X and Y chromosomes. Analyses of transcriptomic data generated by the GTEx Consortium demonstrated that genes with the most significant overexpression in each sex are located on the sex chromosomes.^86^ Similar findings have been generated using data from the BrainSpan consortium, which includes a wider set of brain regions and covers developmental time points. These genes, given their strong sex-bias, are likely playing a role in the sexual differentiation of the brain. However, both the analysis and interpretation of X or Y chromosome-derived sex-biased gene expression require careful consideration.

The presence of the two pseudo-autosomal regions (PAR1 and PAR2) and the process of X inactivation complicates analyses. Outside of the pseudo-autosomal regions, named because of homology with the Y chromosome, one gene copy is generally turned off or inactivated resulting in transcription of a single copy (see Box 3). However, some genes escape X inactivation resulting in higher expression in females, and this can occur in a tissue and cell-specific manner.^101^ Indeed, female-biased gene expression has been used to identify genes potentially escaping from X-inactivation and has been used to explain why X-linked genes with female-biased expression tend to have larger sex effects than either X-linked genes with male-biased expression or other autosomal sex-biased genes.^88^ Furthermore, the enrichment of female-biased gene expression within the younger strata of short arm *P*, again suggests the importance of escape from X chromosome inactivation in driving female-biased gene expression from the X chromosome.

However, not all genes on the X chromosome exhibit sex-biased expression. X chromosome genes of the PAR1 and PAR2 regions as well as other genes outside those regions with a high-degree of Y-linked sequence homology (e.g. *NLGN4X*/*NLGN4Y*, *PRKX*/*PRKY* and *TMSB4X*/*TMSB4Y*) appear to be equally expressed in male and female brains, though with some exceptions (of which *ZFX* is an example).^84^ Yet, the application of standard RNA-sequencing analyses may be contributing to a drop-out of information across this gene set. We note that RNA-sequencing reads mapping to regions with homology in two or more locations of the genome, including those located in the PAR regions of the X chromosome, likely multi-map and would be removed by most standard pipelines. Consequently, alternative strategies for handling multi-mapping reads may be required when considering genes located on the X and Y chromosomes with high sequence homology.

#### Sex-biased transcript usage

Sex-biased differential splicing and transcript use could be another source of sex-genetic biases but is currently understudied, and the largest studies have used array-based methods, which have significant limitations. Trabzuni *et al*.,^84^ found that sex-biases in splicing were widespread, involving 85% (395/448) of all genes with sex-biases in expression of any type across the human brain. Furthermore, analyses of differential exon usage over the course of brain development suggest that sex-biases are most prevalent in prenatal brains, becoming less apparent in adulthood.^83^ Interestingly, in both, the developing and adult human brain, sex-biases in splicing were noted within genes implicated in human brain diseases and known to be sexually dimorphic in presentation or prevalence. Trabzuni *et al*.^84^ identified sex-biases in splicing of *NRXN3*, which has been associated with autism. This gene has two major alternative promoters resulting in the generation of two isoform categories, termed α- and β-neurexins. While levels of α-neurexins were similar between adult males and females, adult males had higher expression of β-neurexins in the thalamus. Further examples of genes with evidence of having sex-biased alternative transcript usage for which the impacted gene is associated with brain disorders are summarized in Table 4. Of note, these examples are from studies that used exon arrays, so highlighting the relatively limited attention this form of sexually dimorphic gene expression has received. Future work using RNA-sequencing data is needed to allow for a more in-depth analysis of transcript usage, and it is arguably quite surprising that this has not already progressed.

**Table 4:** Examples of genes with evidence of sex-biased differential transcript usage in post-mortem brain tissue

Gene	Region	Susceptibility gene for associated brain disorder(s)	Study
*KCNH2*	Brain	Schizophrenia	Kang *et al*., 2011^83^
*NOTCH3*	Brain	Hereditary stroke disorder	Kang *et al*., 2011^83^
*ELN*	Brain	Located in the Williams syndrome critical region	Kang *et al*., 2011^83^
*NLGN4X*	Brain	Autism, moderate X-linked intellectual disability	Kang *et al*., 2011^83^
			Trabzuni *et al*., 2013^84^
*SORL1*	Occipital cortex	Alzheimer’s	Trabzuni *et al*., 2013^84^
*DLG2*	Thalamus	Parkinson’s	Trabzuni *et al*., 2013^84^
*ROBO1*	Putamen	Schizophrenia	Trabzuni *et al*., 2013^84^
*NRXN3*	Thalamus	Autism	Trabzuni *et al*., 2013^84^

#### Sex-biased regulation of gene expression including expression quantitative trait loci (eQTLs)

Although males and females generally express the same transcription factors (TFs), targeting patterns can differ between the sexes, which suggests the existence of sex differences in regulation of gene expression. Brain tissue, along with whole blood, has been found to have amongst the highest proportions of genes, which are expressed in both sexes, but targeted by different TFs.^86^ Across 29 tissues in GTEx, a median of 33% of X chromosome that escape X inactivation were found to be differentially targeted in contrast with 0.3% of autosomal genes.^86^ In this same analysis, while it was noted that 87% of differentially expressed genes were also differentially targeted, 70% of differentially targeted genes were not differentially expressed.^86^ The latter in particular have highlighted the importance of sex differences in transcriptional regulation, which may occur even in the context of similar gene expression across the sexes.

One means of capturing such differences in regulatory networks is through the study of the genetic regulation of gene expression. This is most commonly done through expression quantitative trait loci (eQTLs) analyses and the identification of genetic variants capable of regulating gene expression. Due to limited current data on trans-eQTLs, which require larger sample sizes for detection, this review will focus on cis-eQTLs, defined as eQTLs near (precise range varies) the gene for which it influences expression levels. Across all tissues analysed to date, including brain tissue, evidence for sex-biased eQTLs (sb-eQTLs) has been more limited than might have been expected given the widespread sex-biased gene expression differences observed. For example, while sex-biased expression is detectable in whole blood, the genetic regulation of gene expression in males and females seems to be similar.^102^ In one study, using data from the BIOS consortium and the UK Biobank, the authors found that only 18 genes were found to have significant sb-eQTLs in whole blood out of 9 million genetic variant-gene pairs tested.^103^

Similarly, additional studies on other tissue-types using GTEx data have found limited evidence of sb-eQTLs. Analysis of data from the GTEx Consortium version 8, revealed that out of tens of thousands of cis-eQTLs identified across at least one of 44 different tissues, only 369 sb-eQTLs were found.^87^ These sb-eQTLs were largely tissue-specific and limited to a relatively small number of genes.^88^ Focusing on brain tissue specifically, only eight sb-eQTLs were identified, of which three were detected in the nucleus accumbens, four in the pituitary gland and one in the amygdala. Even within the GTEx study, the numbers of sb-eQTLs were comparatively low. For example, in breast tissue 261 sb-eQTLs were detected in the same study.^88^ Data from the American Brain Expression Consortium and the UKBEC brain banks also found limited evidence of sb-eQTLs in brain tissue.^84^

Other modalities, not mentioned here, such as QTLs associated with protein levels (pQTLs), may also contribute to observed sex differences. Indeed, a recent study assessing the influence of sex on protein abundance and its genetic regulation from more than 1200 human brain proteomes identified 97 putative causal genes with sex-biased protein expression across the major psychiatric and neurodegenerative diseases.^104,105^ The complex combination of some or all these molecular mechanisms likely in part contributes to the observed differences in brain disorders between the sexes.

To summarize, the evidence for sb-eQTLs across tissues, including brain tissue, is currently limited, which may be due to the lack of resolution in the assessed tissue and/or the limited sample size, or it is reflecting the limited sex divergency in regulatory processes.^88,103^ In line with the omnigenic model, stating that in addition to a core set of disease-related genes, it is plausible that sb-eQTLs may be primarily acting in a *trans* rather than a *cis* manner and thus have not been detected due to insufficient power or lack of analysis. Overall, larger sample sizes and fine-resolution single-cell data could help identify sb-eQTLs, and the subsequent pairing of these studies with variant-trait associations could potentially improve our understanding of the roles sb-eQTLs play in sexual differentiation within the brain.

#### To the future

Sex-genotype interactions at the level of transcriptomics can partly explain sex differences observed in brain disorders. When looking at these sex-biased genes, those located on the sex chromosomes stand out since they tend to have the largest effect sizes. However, sex-biased autosomal genes are frequently observed and also contribute to sex differences. Sex-biased expression is a dynamic process, with different genes displaying biases at different developmental stages. Even when a gene is expressed in similar amounts in both sexes, the gene may be targeted by different transcription factors in the sexes, which can result in sex-specific effects. Furthermore, while the current evidence for sb-eQTLs is limited, especially in post-mortem brain tissue, larger sample sizes may help identify certain genes for which sb-eQTLs are the driving factor of sexually dimorphic gene expression. Larger datasets are also needed to better ascertain the role of gene expression in sexual divergence during prenatal development and puberty. Sex-biases can also occur when the relative abundance of transcripts or the levels of gene expression are similar between the sexes as the primary mechanisms driving the relative abundance or levels can differ. Consequently, mechanisms rather than only relative abundance should be explored in future work to more fully understood the sex-genetic landscape.

Moving forward, technical considerations on assessing sex chromosome data, amassing larger sample sizes, and collecting and incorporating information on hormonal states will need to be addressed.

## Discussion

Many brain disorders are complex traits: a result of an interplay between genetic and non-genetic factors, and even monogenic brain disorders have aspects, such as age of onset, which can be modified by interactions with the environment. Differences in prevalence, symptoms, and gene expression in the brain between males and females suggest that sex-genetic interactions can be important factors to investigate in the context of genetic association, gene expression and gene regulation studies for brain disorders. A more in-depth understanding of sex-genetic interactions paves the path towards personalized clinical care that accounts for differences between females and males. A step-by-step roadmap for clinical researchers to perform sex-aware genetic and transcriptomics analyses is currently lacking but is beyond the scope of this review. We note, however, that such guidelines will lead the way to efficiently translate genetic and transcriptomic differences to the clinic. Increasing awareness on the importance of how to best achieve this translation, so that sex differences are routinely incorporated into standard clinical care, is beginning to take place in the context of brain disorders.^3,106^ Increasing awareness is an excellent step in the right direction.

The field is making progress primarily in the study of sex-genetic interactions in the context of brain disorders. Appeals to include chromosome X in GWAS are beginning, but more traction is still needed in this regard. The vast majority of GWAS do not analyse chromosome X variants, and there remain no standards for analyses on this chromosome. Creating agreed-upon guidelines implemented in well-documented tools would promote analyses and enable comparisons across GWAS studies of brain disorders to be done systematically. This is becoming increasingly important as larger sample sizes are becoming available for analyses investigating sex differences in genetic variant-trait associations. These efforts will need to be matched by a similar focus on sex-biases in gene expression, transcript usage and regulation. Changes in post-mortem brain collection strategies, the corresponding meta-data, and the analytical approaches applied will all be required to move forward. We summarize some of the key current gaps and future directions in Table 5.

**Table 5:** Summary of current gaps and future directions

	Current Gaps	Future Directions
Variant-Trait Associations	Low power to identify sex-specific variant-trait associations with small effects	Efforts in increasing cohort sample sizes
	Lack of general chromosome X inclusion in GWAS	Create standardized tools to analyse chromosome X, including PAR regions and handling of chromosome X inactivation
	Limitations on chromosome Y variant calling	Efforts to improve chromosome Y sequence coverage
	Lack of general chromosome Y inclusion in GWAS	Create standards on how to accurately perform associations for chromosome Y
Transcriptomics	Limited sample size of post-mortem brain tissue collections	Efforts in increasing sample sizes, especially for females
	Bias towards collection of male samples	
	Limited access to both pre-menopausal female brain tissues and information on hormonal state	Increase efforts in collection of post-mortem brain tissues at diverse ages
		Collect information about hormonal status
		Development of tools to infer hormonal status (potential using methylation data)
	Multimapping of RNA-seq reads not handled by most standard pipelines and impact on genes located on the sex chromosomes	Awareness of multimapping read issue
		Utilize methods that handle multimapping reads for genes on the X and Y chromosomes with high sequence homology
	Splicing and transcript use data are mostly array-based	Use short- and ultimately long-read RNA sequencing-based approaches to perform splicing and transcript use analyses
	Limited evidence of sex-biased eQTLs across tissues	Efforts in increasing sample sizes to improve power
		Fine resolution single-cell analyses

Interpreting the results of sex-biased variant-trait associations and transcriptional regulation will be complex, with the potential to either over or under interpret findings. Multidisciplinary teams making use of diverse skillsets in epidemiology, computer and data science, biostatistics, cell biology, neurology, endocrinology and psychiatry will be necessary to both identify key insights and translate them clinically.

Nonetheless, we are confident that despite the limitations accounting for and understanding sex-genetic interactions will be pivotal in the push towards personalized medicine and equitable access to accurate diagnosis and treatment between the sexes. Therefore, we encourage researchers across the neurosciences to include both males and females in recruitment strategies, design experiments with sex differences in mind, and to explicitly model sex in genetic and transcriptomics analyses.

## Supplementary Material

fcae192_Supplementary_Data

## Data Availability

The NHGRI-EBI GWAS Catalog data was downloaded from https://www.ebi.ac.uk/gwas/docs/file-downloads.

## References

[1] Demarest TG, Varma VR, Estrada D, et al Biological sex and DNA repair deficiency drive Alzheimer’s disease via systemic metabolic remodeling and brain mitochondrial dysfunction. Acta Neuropathol. 2020;140:25–47. 32333098 10.1007/s00401-020-02152-8 PMC7537767

[2] Labonté B, Engmann O, Purushothaman I, et al Sex-specific transcriptional signatures in human depression. Nat Med. 2017;23:1102–1111. 28825715 10.1038/nm.4386 PMC5734943

[3] Khramtsova EA, Davis LK, Stranger BE. The role of sex in the genomics of human complex traits. Nat Rev Genet. 2019;20:173–190. 30581192 10.1038/s41576-018-0083-1

[4] Wenzl M, McCuskee S, Mossialos E. Commissioning for equity in the NHS: Rhetoric and practice. Br Med Bull. 2015;115:5–17. 26224695 10.1093/bmb/ldv031

[5] Chang DH, Dumanski SM, Ahmed SB. Female sex-specific considerations to improve rigor and reproducibility in cardiovascular research. Am J Physiol Heart Circ Physiol. 2023;324:H279–H287. 36563011 10.1152/ajpheart.00462.2022

[6] Tierney MC, Curtis AF, Chertkow H, Rylett RJ. Integrating sex and gender into neurodegeneration research: A six-component strategy. Alzheimers Dement Transl Res Clin Interv. 2017;3:660–667. 10.1016/j.trci.2017.10.006 PMC5725286 29255793

[7] Bierer BE, Meloney LG, Ahmed HR, White SA. Advancing the inclusion of underrepresented women in clinical research. Cell Rep Med. 2022;3:100553. 35492242 10.1016/j.xcrm.2022.100553 PMC9043984

[8] Friedrich MJ . Depression is the leading cause of disability around the world. JAMA. 2017;317:1517. 10.1001/jama.2017.3826 28418490

[9] Martin LA, Neighbors HW, Griffith DM. The experience of symptoms of depression in men vs women. JAMA Psychiatry. 2013;70:1100. 23986338 10.1001/jamapsychiatry.2013.1985

[10] Marcus SM, Heringhausen JE. Depression in childbearing women: When depression complicates pregnancy. Prim Care Clin Off Pract. 2009;36:151–1ix. 10.1016/j.pop.2008.10.011 PMC2680254 19231607

[11] Sramek JJ, Murphy MF, Cutler NR. Sex differences in the psychopharmacological treatment of depression. Dialogues Clin Neurosci. 2016;18:447–457. 28179816 10.31887/DCNS.2016.18.4/ncutler PMC5286730

[12] Zagni E, Simoni L, Colombo D. Sex and gender differences in central nervous system-related disorders. Neurosci J. 2016;2016:2827090. 27314003 10.1155/2016/2827090 PMC4904110

[13] Ratto AB, Kenworthy L, Yerys BE, et al What about the girls? Sex-based differences in autistic traits and adaptive skills. J Autism Dev Disord. 2018;48:1698–1711. 29204929 10.1007/s10803-017-3413-9 PMC5925757

[14] Gillies GE, Pienaar IS, Vohra S, Qamhawi Z. Sex differences in Parkinson’s disease. Front Neuroendocrinol. 2014;35:370–384. 24607323 10.1016/j.yfrne.2014.02.002 PMC4096384

[15] Martinez-Martin P, Pecurariu CF, Odin P, et al Gender-related differences in the burden of non-motor symptoms in Parkinson’s disease. J Neurol. 2012;259:1639–1647. 22237822 10.1007/s00415-011-6392-3

[16] Mielke MM . Sex and gender differences in Alzheimer’s disease dementia. Psychiatr Times. 2018;35:14–17. 30820070 PMC6390276

[17] Jazin E, Cahill L. Sex differences in molecular neuroscience: From fruit flies to humans. Nat Rev Neurosci. 2010;11:9–17. 20019686 10.1038/nrn2754

[18] McCarthy MM, Arnold AP. Reframing sexual differentiation of the brain. Nat Neurosci. 2011;14:677–683. 21613996 10.1038/nn.2834 PMC3165173

[19] Kamitaki N, Sekar A, Handsaker RE, et al Complement genes contribute sex-biased vulnerability in diverse disorders. Nature. 2020;582:577–581. 32499649 10.1038/s41586-020-2277-x PMC7319891

[20] Levey AS, Stevens LA, Schmid CH, et al A new equation to estimate glomerular filtration rate. Ann Intern Med. 2009;150:604–612. 19414839 10.7326/0003-4819-150-9-200905050-00006 PMC2763564

[21] Anderson TJ, Grégoire J, Hegele RA, et al 2012 update of the Canadian cardiovascular society guidelines for the diagnosis and treatment of dyslipidemia for the prevention of cardiovascular disease in the adult. Can J Cardiol. 2013;29:151–167. 23351925 10.1016/j.cjca.2012.11.032

[22] Spencer CCA, Su Z, Donnelly P, Marchini J. Designing genome-wide association studies: Sample size, power, imputation, and the choice of genotyping chip. PLoS Genet. 2009;5:e1000477. 19492015 10.1371/journal.pgen.1000477 PMC2688469

[23] Stranger BE, Stahl EA, Raj T. Progress and promise of genome-wide association studies for human complex trait genetics. Genetics. 2011;187:367–383. 21115973 10.1534/genetics.110.120907 PMC3030483

[24] The “all of US” research program. N Engl J Med. 2019;381:668–676. 31412182 10.1056/NEJMsr1809937 PMC8291101

[25] Sudlow C, Gallacher J, Allen N, et al UK biobank: An open access resource for identifying the causes of a wide range of Complex diseases of middle and old age. PLoS Med. 2015;12:e1001779. 25826379 10.1371/journal.pmed.1001779 PMC4380465

[26] Nagai A, Hirata M, Kamatani Y, et al Overview of the BioBank Japan project: Study design and profile. J Epidemiol. 2017;27:S2–S8. 28189464 10.1016/j.je.2016.12.005 PMC5350590

[27] Chen Z, Chen J, Collins R, et al China kadoorie biobank of 0.5 million people: Survey methods, baseline characteristics and long-term follow-up. Int J Epidemiol. 2011;40:1652–1666. 22158673 10.1093/ije/dyr120 PMC3235021

[28] Taliun D, Harris DN, Kessler MD, et al Sequencing of 53,831 diverse genomes from the NHLBI TOPMed program. Nature. 2021;590:290–299. 33568819 10.1038/s41586-021-03205-y PMC7875770

[29] Cichon S, Craddock N, Daly M, et al A framework for interpreting genome-wide association studies of psychiatric disorders. Mol Psychiatry. 2009;14:10–17. 19002139 10.1038/mp.2008.126

[30] Bernabeu E, Canela-Xandri O, Rawlik K, Talenti A, Prendergast J, Tenesa A. Sex differences in genetic architecture in the UK biobank. Nat Genet. 2021;53:1283–1289. 34493869 10.1038/s41588-021-00912-0

[31] Nguyen DK, Disteche CM. High expression of the mammalian X chromosome in brain. Brain Res. 2006;1126:46–49. 16978591 10.1016/j.brainres.2006.08.053

[32] Martin J, Khramtsova EA, Goleva SB, et al Examining sex-differentiated genetic effects across neuropsychiatric and behavioral traits. Biol Psychiatry. 2021;89:1127–1137. 33648717 10.1016/j.biopsych.2020.12.024 PMC8163257

[33] Lee S, Abecasis GR, Boehnke M, Lin X. Rare-variant association analysis: Study designs and statistical tests. Am J Hum Genet. 2014;95:5–23. 24995866 10.1016/j.ajhg.2014.06.009 PMC4085641

[34] Munafò MR, Tilling K, Taylor AE, Evans DM, Smith GD. Collider scope: When selection bias can substantially influence observed associations. Int J Epidemiol. 2018;47:226–235. 29040562 10.1093/ije/dyx206 PMC5837306

[35] Delgado-Rodriguez M . Bias. J Epidemiol Community Health (1978). 2004;58:635–641. 10.1136/jech.2003.008466 PMC1732856 15252064

[36] Strother E, Lemberg R, Stanford SC, Turberville D. Eating disorders in men: Underdiagnosed, undertreated, and misunderstood. Eat Disord. 2012;20:346–355. 22985232 10.1080/10640266.2012.715512 PMC3479631

[37] Sonneville KR, Lipson SK. Disparities in eating disorder diagnosis and treatment according to weight status, race/ethnicity, socioeconomic background, and sex among college students. Int J Eat Disord. 2018;51:518–526. 29500865 10.1002/eat.22846

[38] Silveira PP, Pokhvisneva I, Howard DM, Meaney MJ. A sex-specific genome-wide association study of depression phenotypes in UK biobank. Mol Psychiatry. 2023;28:2469–2479. 36750733 10.1038/s41380-023-01960-0 PMC10611579

[39] Wendt FR, Pathak GA, Singh K, et al Sex-Specific genetic and transcriptomic liability to neuroticism. Biol Psychiatry. 2023;93:243–252. 36244801 10.1016/j.biopsych.2022.07.019 PMC10508260

[40] Blokland GAM, Grove J, Chen C-Y, et al Sex-Dependent shared and nonshared genetic architecture across mood and psychotic disorders. Biol Psychiatry. 2022;91:102–117. 34099189 10.1016/j.biopsych.2021.02.972 PMC8458480

[41] Mitra I, Tsang K, Ladd-Acosta C, et al Pleiotropic mechanisms indicated for sex differences in autism. PLoS Genet. 2016;12:e1006425. 27846226 10.1371/journal.pgen.1006425 PMC5147776

[42] Blauwendraat C, Heilbron K, Vallerga CL, et al Parkinson’s disease age at onset genome-wide association study: Defining heritability, genetic loci, and α-synuclein mechanisms. Mov Disord. 2019;34:866–875. 30957308 10.1002/mds.27659 PMC6579628

[43] Dumitrescu L, Barnes LL, Thambisetty M, et al Sex differences in the genetic predictors of Alzheimer’s pathology. Brain. 2019;142:2581–2589. 31497858 10.1093/brain/awz206 PMC6736148

[44] Deming Y, Dumitrescu L, Barnes LL, et al Sex-specific genetic predictors of Alzheimer’s disease biomarkers. Acta Neuropathol. 2018;136:857–872. 29967939 10.1007/s00401-018-1881-4 PMC6280657

[45] Eissman JM, Dumitrescu L, Mahoney ER, et al Sex differences in the genetic architecture of cognitive resilience to Alzheimer’s disease. Brain. 2022;145:2541–2554. 35552371 10.1093/brain/awac177 PMC9337804

[46] Shvetsova E, Sofronova A, Monajemi R, et al Skewed X-inactivation is common in the general female population. Eur J Hum Genet. 2019;27:455–465. 30552425 10.1038/s41431-018-0291-3 PMC6460563

[47] Sidorenko J, Kassam I, Kemper KE, et al The effect of X-linked dosage compensation on complex trait variation. Nat Commun. 2019;10:3009. 31285442 10.1038/s41467-019-10598-y PMC6614401

[48] Johnson KE, Voight BF. Patterns of shared signatures of recent positive selection across human populations. Nat Ecol Evol. 2018;2:713–720. 29459708 10.1038/s41559-018-0478-6 PMC5866773

[49] Veeramah KR, Gutenkunst RN, Woerner AE, Watkins JC, Hammer MF. Evidence for increased levels of positive and negative selection on the X chromosome versus autosomes in humans. Mol Biol Evol. 2014;31:2267–2282. 24830675 10.1093/molbev/msu166 PMC4137703

[50] Nath SK, Han S, Kim-Howard X, et al A nonsynonymous functional variant in integrin-αM (encoded by ITGAM) is associated with systemic lupus erythematosus. Nat Genet. 2008;40:152–154. 18204448 10.1038/ng.71

[51] Nguyen DK, Disteche CM. Dosage compensation of the active X chromosome in mammals. Nat Genet. 2006;38:47–53. 16341221 10.1038/ng1705

[52] McKusick-Nathans Institute of Genetic Medicine, Johns Hopkins University (Baltimore, MD). Online Mendelian Inheritance in Man, OMIM. Accessed 28 August 2023. https://www.omim.org

[53] Ross MT, Grafham D V, Coffey AJ, et al The DNA sequence of the human X chromosome. Nature. 2005;434:325–337. 15772651 10.1038/nature03440 PMC2665286

[54] Wise AL, Gyi L, Manolio TA. EXclusion: Toward integrating the X chromosome in genome-wide association analyses. Am J Hum Genet. 2013;92:643–647. 23643377 10.1016/j.ajhg.2013.03.017 PMC3644627

[55] Sun L, Wang Z, Lu T, Manolio TA, Paterson AD. Exclusionary: 10 years later, where are the sex chromosomes in GWASs? Am J Hum Genet. 2023;110:903–912. 37267899 10.1016/j.ajhg.2023.04.009 PMC10257007

[56] Miga KH, Koren S, Rhie A, et al Telomere-to-telomere assembly of a complete human X chromosome. Nature. 2020;585:79–84. 32663838 10.1038/s41586-020-2547-7 PMC7484160

[57] Rhie A, Nurk S, Cechova M, et al The complete sequence of a human Y chromosome. Nature. 2023;621:344–354. 37612512 10.1038/s41586-023-06457-y PMC10752217

[58] Leale TP, French-Kwawu JN, Gouveia MH, et al X-Chromosome Association Study in Latin American cohorts identifies new loci in Parkinson's disease. Mov Disord: Off J Mov Disord Soc. 2023;38(9):1625–1635. 10.1002/mds.29508 PMC10524402 37469269

[59] Patsopoulos NA, Baranzini SE, Santaniello A, et al Multiple sclerosis genomic map implicates peripheral immune cells and microglia in susceptibility. Science (1979). 2019;365:eaav7188–eaav7188. 10.1126/science.aav7188 PMC7241648 31604244

[60] Lam M, Chen C-Y, Li Z, et al Comparative genetic architectures of schizophrenia in east Asian and European populations. Nat Genet. 2019;51:1670–1678. 31740837 10.1038/s41588-019-0512-x PMC6885121

[61] Wang J, Yu R, Shete S. X-chromosome genetic association test accounting for X-inactivation, skewed X-inactivation, and escape from X-inactivation. Genet Epidemiol. 2014;38:483–493. 25043884 10.1002/gepi.21814 PMC4127090

[62] Gao F, Chang D, Biddanda A, et al XWAS: A software toolset for genetic data analysis and association studies of the X chromosome. J Hered. 2015;106:666–671. 26268243 10.1093/jhered/esv059 PMC4567842

[63] Chen B, Craiu R V, Sun L. Bayesian model averaging for the X-chromosome inactivation dilemma in genetic association study. Biostatistics. 2020;21:319–335. 30247537 10.1093/biostatistics/kxy049

[64] Clayton D . Testing for association on the X chromosome. Biostatistics. 2008;9:593–600. 18441336 10.1093/biostatistics/kxn007 PMC2536723

[65] Purcell S, Neale B, Todd-Brown K, et al PLINK: A tool set for whole-genome association and population-based linkage analyses. Am J Hum Genet. 2007;81:559–575. 17701901 10.1086/519795 PMC1950838

[66] Carrel L, Willard HF. X-inactivation profile reveals extensive variability in X-linked gene expression in females. Nature. 2005;434:400–404. 15772666 10.1038/nature03479

[67] Slavney A, Arbiza L, Clark AG, Keinan A. Strong constraint on human genes escaping X-inactivation is modulated by their expression level and breadth in both sexes. Mol Biol Evol. 2016;33:384–393. 26494842 10.1093/molbev/msv225 PMC4751236

[68] Tukiainen T, Villani AC, Yen A, et al Landscape of X chromosome inactivation across human tissues. Nature. 2017;550:244–248. 29022598 10.1038/nature24265 PMC5685192

[69] Zito A, Davies MN, Tsai PC, et al Heritability of skewed X-inactivation in female twins is tissue-specific and associated with age. Nat Commun. 2019;10:5339. 31767861 10.1038/s41467-019-13340-w PMC6877649

[70] Zhou W, Nielsen JB, Fritsche LG, et al Efficiently controlling for case-control imbalance and sample relatedness in large-scale genetic association studies. Nat Genet. 2018;50:1335–1341. 30104761 10.1038/s41588-018-0184-y PMC6119127

[71] Loh PR, Tucker G, Bulik-Sullivan BK, et al Efficient Bayesian mixed-model analysis increases association power in large cohorts. Nat Genet. 2015;47:284–290. 25642633 10.1038/ng.3190 PMC4342297

[72] Mbatchou J, Barnard L, Backman J, et al Computationally efficient whole-genome regression for quantitative and binary traits. Nat Genet. 2021;53:1097–1103. 34017140 10.1038/s41588-021-00870-7

[73] Lee J, Pinares-Garcia P, Loke H, Ham S, Vilain E, Harley VR. Sex-specific neuroprotection by inhibition of the Y-chromosome gene, *SRY*, in experimental Parkinson’s disease. Proc Natl Acad Sci USA. 2019;116:16577–16582. 31371505 10.1073/pnas.1900406116 PMC6697880

[74] Hallast P, Ebert P, Loftus M, et al Assembly of 43 human Y chromosomes reveals extensive complexity and variation. Nature. 2023;621:355–364. 37612510 10.1038/s41586-023-06425-6 PMC10726138

[75] Parker K, Mesut Erzurumluoglu A, Rodriguez S. The Y chromosome: A complex locus for genetic analyses of complex human traits. Genes (Basel). 2020;11(1273):1–19. 10.3390/genes11111273 PMC7693691 33137877

[76] Grenn FP, Makarious MB, Bandres-Ciga S, et al Analysis of Y chromosome haplogroups in Parkinson’s disease. Brain Commun. 2022;4:fcac277. 36387750 10.1093/braincomms/fcac277 PMC9665271

[77] Smith GD, Ebrahim S. “Mendelian randomization”: Can genetic epidemiology contribute to understanding environmental determinants of disease? Int J Epidemiol. 2003;32:1–22. 12689998 10.1093/ije/dyg070

[78] Anderson CA, Pettersson FH, Clarke GM, Cardon LR, Morris AP, Zondervan KT. Data quality control in genetic case-control association studies. Nat Protoc. 2010;5:1564–1573. 21085122 10.1038/nprot.2010.116 PMC3025522

[79] Marees AT, de Kluiver H, Stringer S, et al A tutorial on conducting genome-wide association studies: Quality control and statistical analysis. Int J Methods Psychiatr Res. 2018;27:e1608. 29484742 10.1002/mpr.1608 PMC6001694

[80] Khramtsova EA, Wilson MA, Martin J, et al Quality control and analytic best practices for testing genetic models of sex differences in large populations. Cell. 2023;186:2044–2061. 37172561 10.1016/j.cell.2023.04.014 PMC10266536

[81] Miller JA, Ding S-L, Sunkin SM, Smith KA, Ng L, Szafer A, et al Transcriptional landscape of the prenatal human brain. Nature. 2014;508:199–206. 24695229 10.1038/nature13185 PMC4105188

[82] Shi L, Zhang Z, Su B. Sex biased gene expression profiling of human brains at Major developmental stages. Sci Rep. 2016;6:21181. 26880485 10.1038/srep21181 PMC4754746

[83] Kang HJ, Kawasawa YI, Cheng F, et al Spatio-temporal transcriptome of the human brain. Nature. 2011;478:483–489. 22031440 10.1038/nature10523 PMC3566780

[84] Trabzuni D, Ramasamy A, Imran S, et al Widespread sex differences in gene expression and splicing in the adult human brain. Nat Commun. 2013;4:2771. 24264146 10.1038/ncomms3771 PMC3868224

[85] Ardlie KG, Deluca DS, Segrè AV, Sullivan TJ, Young TR, Gelfand ET, et al The Genotype-Tissue Expression (GTEx) pilot analysis: Multitissue gene regulation in humans. Science (1979). 2015;348:648–660. 10.1126/science.1262110 PMC4547484 25954001

[86] Lopes-Ramos CM, Chen C-Y, Kuijjer ML, et al Sex differences in gene expression and regulatory networks across 29 human tissues. Cell Rep. 2020;31:107795. 32579922 10.1016/j.celrep.2020.107795 PMC7898458

[87] Aguet F, Barbeira AN, Bonazzola R, et al The GTEx consortium atlas of genetic regulatory effects across human tissues. Science (1979). 2020;369:1318–1330. 10.1126/science.aaz1776 PMC7737656 32913098

[88] Oliva M, Munoz-Aguirre M, Kim-Hellmuth S, et al The impact of sex on gene expression across human tissues. Science. 2020; 369: eaba3066. 32913072 10.1126/science.aba3066 PMC8136152

[89] Bennett DA, Buchman AS, Boyle PA, Barnes LL, Wilson RS, Schneider JA. Religious orders study and rush memory and aging project. J Alzheimers Dis. 2018;64:S161–S189. 29865057 10.3233/JAD-179939 PMC6380522

[90] Xia Y, Dai R, Wang K, Jiao C, Zhang C, Xu Y, et al Sex-differential DNA methylation and associated regulation networks in human brain implicated in the sex-biased risks of psychiatric disorders. Mol Psychiatry. 2021;26:835–848. 30976086 10.1038/s41380-019-0416-2 PMC6788945

[91] Douglas Mental Health University Institute . The Douglas-Bell Canada Brain Bank. Douglas Bell Canada Brain Bank; 2023.

[92] Akbarian S, Liu C, Knowles JA, et al The PsychENCODE project. Nat Neurosci. 2015;18:1707–1712. 26605881 10.1038/nn.4156 PMC4675669

[93] Gandal MJ, Zhang P, Hadjimichael E, et al Transcriptome-wide isoform-level dysregulation in ASD, schizophrenia, and bipolar disorder. Science (1979). 2018;362:eaat8127–eaat8127. 10.1126/science.aat8127 PMC6443102 30545856

[94] Fromer M, Roussos P, Sieberts SK, et al Gene expression elucidates functional impact of polygenic risk for schizophrenia. Nat Neurosci. 2016;19:1442–1453. 27668389 10.1038/nn.4399 PMC5083142

[95] Hoffman GE, Bendl J, Voloudakis G, et al CommonMind consortium provides transcriptomic and epigenomic data for schizophrenia and bipolar disorder. Sci Data. 2019;6:180. 31551426 10.1038/s41597-019-0183-6 PMC6760149

[96] Weickert CS, Elashoff M, Richards AB, et al Transcriptome analysis of male–female differences in prefrontal cortical development. Mol Psychiatry. 2009;14:558–561. 19455171 10.1038/mp.2009.5

[97] Kwon Y-S, Garcia-Bassets I, Hutt KR, et al Sensitive ChIP-DSL technology reveals an extensive estrogen receptor α-binding program on human gene promoters. Proc Natl Acad Sci USA. 2007;104:4852–4857. 17360330 10.1073/pnas.0700715104 PMC1821125

[98] Ochoa D, Hercules A, Carmona M, et al The next-generation open targets platform: Reimagined, redesigned, rebuilt. Nucleic Acids Res. 2023;51:D1353–D1359. 36399499 10.1093/nar/gkac1046 PMC9825572

[99] Macpherson JN, Murray A. Development of genetic testing for fragile X syndrome and associated disorders, and estimates of the prevalence of FMR1 expansion mutations. Genes Basel. 2016;7(110):1–15. 10.3390/genes7120110 PMC5192486 27916885

[100] Dibbens LM, Tarpey PS, Hynes K, et al X-linked protocadherin 19 mutations cause female-limited epilepsy and cognitive impairment. Nat Genet. 2008;40:776–781. 18469813 10.1038/ng.149 PMC2756413

[101] Zito A, Roberts AL, Visconti A, et al Escape from X-inactivation in twins exhibits intra- and inter-individual variability across tissues and is heritable. PLoS Genet. 2023;19:e1010556. 36802379 10.1371/journal.pgen.1010556 PMC9942974

[102] Kassam I, Lloyd-Jones L, Holloway A, et al Autosomal genetic control of human gene expression does not differ across the sexes. Genome Biol. 2016;17:248. 27908293 10.1186/s13059-016-1111-0 PMC5134098

[103] Porcu E, Claringbould A, Weihs A, et al Limited evidence for blood eQTLs in human sexual dimorphism. Genome Med. 2022;14:89. 35953856 10.1186/s13073-022-01088-w PMC9373355

[104] Wingo AP, Liu Y, Gerasimov ES, et al Sex differences in brain protein expression and disease. Nat Med. 2023;29:2224–2232. 37653343 10.1038/s41591-023-02509-y PMC10504083

[105] Wingo TS, Liu Y, Gerasimov ES, et al Shared mechanisms across the major psychiatric and neurodegenerative diseases. Nat Commun. 2022;13:4314. 35882878 10.1038/s41467-022-31873-5 PMC9325708

[106] Riecher-Rössler A . Oestrogens, prolactin, hypothalamic-pituitary-gonadal axis, and schizophrenic psychoses. Lancet Psychiatry. 2017;4:63–72. 27856396 10.1016/S2215-0366(16)30379-0

