# Supplementary material for: Leveraging sex-genetic interactions to understand brain disorders: recent advances and current gaps
Source: Brain Commun. 2024 Jun 3;6(3):fcae192. doi: 10.1093/braincomms/fcae192 (PMC11184352; doi:10.1093/braincomms/fcae192)
Supplement: fcae192_Supplementary_Data [file fcae192_supplementary_data.pdf]

Supplementary Material

Supplementary Table 1. Sex chromosome support for a non-exhaustive list of commonly used genetic association testing software.

Software	Reference	Chromosome X	Chromosome Y
PLINK	Purcell et al. 2007	Option 1: Female-only analysis Option 2: Use all samples in the regression, and include sex as a covariate	Treat heterozygous Y genotypes as missing
SAIGE	Zhou et al. 2018	Handled the same as autosomal variants	Option 1: Run Step 1 (relatedness matrix construction and variance ratio) for males only, and then run Step 2 (association testing) for chromosome Y for males. Option 2: Use Step 1 output with all samples, and in Step 2, set flag --IsDropMissingDosages= TRUE to remove missing dosages on the Y
Bolt-LMM	Loh et al. 2015	Handled the same as autosomal variants	Support is not implemented
Regenie	Mbatchou et al. 2021	Males should be coded as diploid so that their genotypes are 0/2	Support is not implemented
